# The Sanitary State of the Army in India

**Published:** 1863-10

**Authors:** 


### Art. VI.—THE SANITARY STATE OF THE ARMY IN INDIA.

Whatever position history may accord to the military events of the Crimean War, little doubt can be entertained that to the results of the campaign before Sebastopol the sanitary regeneration of the British soldier is to be traced. The great catastrophe of the winter of 1854 has been paralleled by other disasters to British forces, arising from like causes, as at Walcheren, Rangoon, and in China. The bitter truths taught by that catastrophe were neither novel nor unknown. They had been iterated and reiterated, for a century or more, in words as emphatic as the language possessed, by army medical men. The pages of Pringle, Robert Jackson, William Fergusson, Hennen, and Guthrie bristle with them. But precept and example—nay, even disaster and disgrace—alike failed to impress Government and the military authorities with the momentous character of these truths. The public, also, in a great measure ignorant of the needless waste of life and strength of our army in peace, and regarding this waste as a necessary incident in war, looked solely to the fighting efficiency of the forces in number and armament, and found ample consolation in success for the cost, however terrible, at which it might have been purchased. The soldier was, on all sides, held to be a mere machine—a living, sentient, self-acting one truly, but still a machine. If this machine could be trained to a thorough knowledge of weapons, and to act in full co-operation with other like machines, what more was needed? Its efficiency was estimated by its destructiveness, and its destructiveness by the perfectness to which it could be drilled to combined action, and its skill in wielding the means of offence. Certainly the machine required feeding, but this was a question of quantity merely. It had to be clad, but what more could be needed than to clothe it prettily? It had to be housed, but shelter from the wind and the rain was surely sufficient. It was self-acting, and apt to indulge in erratic movements, neither conducive to its own welfare nor that of the community—well, there were the provost-marshal and the chaplain as monitors. It was liable to fall out of order, but, if such were its fate, there were the hospital and the doctor. Truly, as it would seem, a well-ordered and well-found machine! But the incidents of the sad winter of 1854 taught another, and widely different story. Then England learnt for the first time how strangely it had misapprehended the requirements of the soldier.

By the electric telegraph, the facility of correspondence given by steam-power, and the marvellous intelligence of the daily press, the most intimate *life* (so to speak) of the Crimean army was laid open to the English public, from the time of its leaving these shores, until the time of its return. Every act, every movement, every shortcoming, every achievement, whatever most concerned the welfare of the soldier and the honour of the kingdom, all was set forth, at the moment, with a completeness and power of description altogether unparalleled in contemporary records of war. The citizen at home, sitting in peace by his fireside, possessed a more accurate knowledge of the progress of events at the seat of war, in their entirety, than the man who was engaged in the struggle.

The heart swelled as the thoughts dwelt on the gallant army which left England for the East, perfect in the matured powers of the men, in their admirable training and superb and lavish equipment. How eagerly the fancy followed the troops day by day, almost hour by hour, from point to point of their destined course, and from camp to camp! How vividly it pictured to itself every event of the soldier's life to the minutest detail—for not an item was lacking from the wondrously graphic narrations of journalists—at Gallipoli, at Varna, and in the great beleaguerment which crowned and ended the war. But the force had barely landed on a foreign soil, when an uneasy consciousness that all was not as it should be began to possess the public mind. The soldier did not appear to be quite at home under canvas; neither officers nor men exhibited any very great aptitude for campaigning; there was a painful degree of bewilderment in the commissariat; and stores were often missing in an incomprehensible manner, or were not forthcoming when most required, or were needlessly difficult of access. Contrasted with the French forces, the campaigning qualifications of our troops presented a somewhat sorry figure. It began to be suspected that the training to which British soldiers were subjected, admirable though it might be for the parade-ground, and probably for the tug of actual strife, did not initiate them into the habits fitted for active warfare and a life under canvas—did not fit them, in short, for that very phase of life which of all others is peculiar to the soldier. The suspicion grew with the progress of the war, gaining strength from the events at Varna and on the beach near Eupatoria, the night after the descent upon the Crimea. The horrible dead-lock at Scutari quickly followed, and doubt was at an end. With amazed indignation the public saw an utter absence of organization in the general hospitals, department clashing with department in hopeless confusion, and official routine setting at nought the dearest interests of the soldier and honour of the country. It witnessed the wounded of Alma, of Balaclava, and of Inkermann, and the rapidly increasing sick from the camp before Sebastopol, crowded together without order or decency, and wanting even the sheerest necessities amidst an apparent profusion of stores, and within cannon-shot of a great city. It witnessed these gallant men rotting away amidst revolting filth and neglect; the vast buildings in which they were housed converted into foul pest-houses; the medical staff helpless amidst the trammels of insensate regulations; and the military authorities deaf to remonstrance, and placidly replying to all protests that they had no official information that the state of things was such as the public press represented it to be.

Then the indignation of the people broke forth. It intervened with a force which would not be said nay to, between the sick and wounded and the authorities. From its abundance the nation poured forth whatever was needed to give its maimed and helpless soldiers ease, comfort, and the hope of life. The *Times*, true to its great mission, organized this spontaneous outbreak of public feeling; and FLORENCE NIGHTINGALE appeared upon the scene, and like the glorious angel, who, descending at the gates of Dis, lit up the murky air with a splendid radiance and dispelled the shadows from Dante's countenance, so she dispelled the hideous gloom which had gathered around the sick and the dying at Scutari, and extended its dark shadow to every hearth in the kingdom, from the palace to the cottage.

But the worst was yet to come. The pitiless winter fell upon the Crimea. Little had been rightly foreseen; little fittingly provided for by the military authorities. But for the indomitable courage of officers and men—a courage which, alas! was most conspicuously displayed in contending against needlessly created evils—the entire extinction of the army and a disgraceful ending of the campaign could hardly have been prevented. A mile and a half of neglected road, torn up by ammunition waggons and heavy guns, and converted into an almost impassable slough by the first rains, reduced the troops to the verge of starvation, while from the heights on which they kept weary watch and ward could be seen the tall masts of ships deeply laden with all things which might supply their great needs. Disaster complicated the want of foresight, and the rigours of winter found the men in summer garments, and imperfectly sheltered beneath tents which afforded little protection against cold and wet. Scanty rations were made still more scanty by the ignorance of all ranks in what manner best to cook and economize their food. Nay, the ignorance in this respect was so great, that not unfrequently it happened that the men suffered all the evils of privation from their inability to turn to proper account a sufficient ration. Fuel, when scarce, was wasted egregiously by the want of knowledge how most economically to use it. The encampments became diffused cesspools, partly from the enforced neglect of overtaxed powers, partly from the absence of all definite sanitary regulations. The miseries of the sick were increased by the want of every comfort, and even by a deficiency of the most necessary drugs, tons of which were lying idle and inaccessible from some insensate official caprice, or from being buried beneath irremovable cargoes in the holds of transports. No one was responsible for a fitting adoption of the resources at hand to the wants of the soldier. Under the combined influence of excessive fatigue, insufficient rations, salt-meat, and extreme scarcity of vegetables, scurvy quickly appeared among the troops and heightened their wretchedness. Herds of cattle abounded on the south coasts of the Black Sea, but the commissariat pleaded insufficiency of transport. An illimitable supply of vegetables might be obtained within a three days' voyage, but it was no part of the commissariat's duty to provide vegetables, as they did not form a portion of the ordinary ration. The authorities at home tantalized the troops by sending out green coffee; the authorities at the seat of war did not conceive that the rectification of the mischievous error was one of their functions. While the organization of the French army permitted the issue of soft bread to our allies from the beginning of the campaign, the scurvy-stricken British forces were tortured with hard biscuit, which their spongy and bleeding gums would hardly permit them to gnaw. Nay more, with an apathy almost incredible, the higher powers suffered large quantities of lime-juice, which would have mitigated the sufferings of the men, to lay for weeks unheeded in the commissariat stores.

The different departments, indeed, strove to adopt the routine of peace to the contingencies of war. It was no part of their duty to modify the standing regulations of the home service. The welfare of the soldier sunk into insignificance before the requirements of official custom. The lives of the men were weighed against vouchers and returns, and found wanting. The authorities at home, moreover, did not work in perfect accord with their subordinates abroad. Every one was in fault, and yet no one was to blame. Forty years of peace had given rise to a military system unfitted for war, and experience had once more to be gathered at the cost of a great and gallant army.

Happily for the nation, the then Secretary-at-War, Mr. Sidney Herbert, afterwards Lord Herbert of Lea, rightly appreciated the true significance of the great disaster which darkened his period of office. Thenceforward he devoted himself, at the expense of labours which, alas! too quickly undermined his physical powers, and shortened his life, to those inquiries into the sanitary condition of the soldier, which ended in the regeneration of the army at home and in the colonies, and which are about to lead to the same result for the army in India.

Under the immediate influence of the catastrophe which had befallen the army before Sebastopol, and of the unrestrained indignation of the public, Royal Commissioners were sent to the seat of war to investigate the state of the Hospitals, the Supplies, and the Camps. The result of these inquiries conclusively showed that the sufferings of the troops were less dependent upon the contingencies of a winter campaign than upon the defective organization of the different departments of the military service, and the ignorance of both officers and men of the actual requirements of campaigning. The suggestions of the Commissioners, which, to a great extent, had been anticipated by the correspondents of the public press, and which were obvious from the moment of the forces landing in the Crimea, had, when carried into effect, the almost immediate result of redeeming the army from the slough of misery into which it had been plunged. The hospitals were quickly reduced to order and efficiency, the health of the men speedily rallied, and the great gaps made by death were filled up. From month to month the physical vigour of the troops improved, notwithstanding the harassing duties of the siege, and the evil sanitary condition in which, at the best, they were too frequently of necessity placed; and before the end of the war the remarkable spectacle was presented of an army maintaining a higher degree of health in the field, in presence of the enemy, than when comfortably housed in barracks at home during peace.

After the termination of the war, that more complete investigation of the internal economy of the army was commenced, which the events of 1854 had shown to be so highly requisite. In 1857 a Royal Commission was appointed, under the presidency of Lord Herbert, to inquire into the regulations affecting the sanitary condition of the forces, the organization of military hospitals, and the treatment of the sick and wounded. The investigations of this Commission unmistakeably proved that the disaster of 1854 was the natural and necessary result of the sanitary organization, or rather disorganization, of the army. The evils which had assumed proportions so gigantic during the early period of the Crimean War, were found to exist in a very appreciable degree in a chronic form, during peace, among the troops at home. Although soldiers were picked men, apparently comfortably housed, abundantly fed, well clothed, in every respect well-cared for, and free from the anxieties which beset the struggle for existence in civil life, it was discovered that their chance of life was but half that of civilians of their own age, of all classes, and under all circumstances, and one-third less than the said civilians, when these were placed under circumstances the most unhealthy. The explanation of this strange anomaly was not difficult of detection. If the soldier was sheltered under a good roof, and behind stout walls, yet he and his fellows were so closely herded together, his quarters were constructed with so little attention to the commonest sanitary considerations, and the means of personal cleanliness were so imperfect, that the maintenance of a degree of health befitting his age and apparent position was an impossibility. Every barracks in the kingdom, almost without exception, presented that neglect of sanitary precautions, that tendency to overcrowding, and that indifference, and even complete neglect, of the needful means of personal cleanliness, which were the first conspicuous evils at Scutari, and which, baneful to the men when in health, exercised the most deadly influence over the sick and wounded. If the soldier were seemingly abundantly fed, yet his ration was imperfect; he was left to his own fancy in supplying the articles of food necessary to complete it, and his means of cooking were so defective that it was impossible for him to adopt any but the most extravagant and wasteful modes of preparing his food. He was debarred from a knowledge of the ration upon which he would have to subsist in the field; or from acquiring any acquaintance with the best, most economical, readiest, and most savoury way of cooking it. The absence of a complete ration implied the absence of a commissariat, and of officers tutored in the alimentary requirements of troops, or whose experience of supplies extended beyond the necessities of a badly conceived peace establishment. It also confirmed the soldier in the extravagant and wasteful habits of cooking, and limited range of articles of food, which are unhappily so common among the classes from which he is chiefly derived, and unfitted him for that ready adaptation to the culinary needs of a campaign, particularly when food might be scanty. These evils were largely accountable for the miseries of 1854. If the soldier were apparently well clad, it was not the less true that his clothing was ill-fitted for the exigencies of active war. If he were strictly disciplined, it was also true that his hours of idleness were so many, and so little provision had been made to turn them to good account, that he was the victim of dissipation and vice. Intemperance was one of the gravest evils during the Crimean War, and the military authorities pandered to the habit in an inexplicable manner by the facilities they afforded to the men of procuring the most poisonous spirits. If he were well tended when sick, it is certain that military hospitals had many imperfections, and that the provision for his sickness was adapted only to times of peace.* In short, immediately prior to the Russian War, there was neither sanitary organization nor regulation, properly so called, in the army, and the warlike training of the soldier was limited entirely to proficiency in the use of weapons and combined action. This was the root of the disaster of 1854.

The Royal Commission, also appointed in 1857, for improving the sanitary condition of Barracks and Military Hospitals, in their Report, published in 1861, fully confirmed the conclusions of the Sanitary Commission on the defective sanitary condition of barracks and hospitals.

The investigation into the sanitary condition of the army remained, however, incomplete, until it included the British troops in India. Accordingly, in 1859, a Royal Commission was appointed to inquire into the Sanitary State of the Indian Army, Lord Herbert again presiding. The chairman's health unhappily gave way, and his lamented death occurred before the inquiry was completed. Lord Stanley succeeded Lord Herbert as chairman, and he presided over the Commission until it terminated its labours. Another member of the Commission, Mr. Alexander, the Director-General of the Army Medical Department, was also lost by death. The Commission reported the result of its long and laborious labours in the course of the past quarter. The members of the Committee, when its duties terminated, were Lord Stanley; Sir Proby P. Cautley, K.C.B., Member of the Council of India; Sir J. Ranald Martin, C.B., F.R.S., Physician to the Council of India; J. B. Gibson, M.D., Director-General of the Army Medical Department; Colonel E. N. Greathed, C.B.; Dr. Farr, M.D., F.R.S., D.C.L.; and J. Sutherland, M.D.

Those who were familiar with the writings of Indian medical authorities, would be prepared to find in the three Presidencies a repetition of the sanitary evils disclosed in barracks, hospitals, and the internal economy of the army at home, but exaggerated by a torrid climate. Those who had not access to these authorities, but who knew the rigour of military routine, would hardly anticipate either better sanitary arrangements or a better system

---

* "The Report of the Royal Commission," says Lord Herbert, in a letter prefixed to the New Code of Medical Regulations for the Army, "has shown the high rate of mortality from disease existing among the troops at all times; but more especially during war—the defective condition of military hospitals—the absence of any means of organizing general hospitals in time of war—the want of any method by which the improvements recently introduced for the protection of health in civil life can be rendered available in barracks, camps, and hospitals—and the great loss of life arising from these defects during the late war with Russia."

of sanitary organization among our army in the distant East than among the forces in the British Islands, notwithstanding that the autocratic powers of the Indian Government afforded greater facility for carrying out both the one and the other. The Report of the Commission confirms the worst forebodings.

We are told that the stations in India have, as a rule, been selected without any reference to health, and that very commonly they are so situated as to expose the troops to local causes of disease of the gravest character. Again, the Report states:—

"None of the stations have any sub-soil drainage; and there are no other means of removing the rainfall except surface-gutters. The ground about the lines is often broken into pits and hollows, filled with stagnant water, or is traversed by unwholesome ravines or nullahs. In certain states of the weather and wind, nuisance is experienced in the lines from these causes, and from the foul state of neighbouring native dwellings. Many of the older stations are irregularly built; and the buildings are arranged so as to interfere with each other's ventilation." (p. 161.*)

It is impossible, moreover, to disconnect the sanitary state of the stations from that of the native towns and bazaars, "because not only is a part of the soldier's time spent in these places, but the mere fact of their proximity to European barracks must necessarily exercise an injurious influence on the healthiness of both barracks and hospitals, if the native dwellings are in an unwholesome condition." (p. 77.) Every military station in India has its bazaars, mostly in close proximity to the European lines, and containing a native population which always bears a very high proportion to that of the European troops at the station. Bangalore, for example, has accommodation for 1700 European, and 2600 native troops. The native population within the cantonment, three-fourths of whom live in the bazaar close to the European infantry barrack, numbers 124,000. "It is impossible," says the Report, "to separate the question of health, as it relates to troops, from the sanitary condition of the native population; especially as it regards the occurrence of epidemics, which, whenever they occur among natives, indicate a condition of matters dangerous in the highest degree to the troops in the neighbourhood." (p. 77.)

The recognition of this great truth is of fundamental importance in the sanitary government of the Indian army. The habits of the natives, unless closely watched, are singularly filthy; and the sanitary condition of towns and bazaars is thus summed up in the Report:—

* The references are to the 8vo Blue Book, containing the Report of the Commission, and a *précis* of the evidence. By an excellent arrangement, these have been published in a separate form. The Report with evidence forms two prodigious and unmanageable volumes.

"The towns and bazaars, in the vicinity of lines, are in the worst possible sanitary state—undrained, unpaved, badly cleansed, often teeming with offensive and dangerous nuisances; with tanks, pools, and badly-made surface-gutters, containing filth and foul water; the area overcrowded with houses, put up without order or regularity; the external ventilation obstructed, and the houses over-crowded with people; no public latrines, and every spare plot of ground covered with filth in consequence; no water-supply, except what is obtained from bad shallow wells, and unwholesome or doubtful tanks. These towns and bazaars are the earliest seats of epidemics, especially of cholera, before their ravages extend to the European troops in the vicinity." (p. 161.)

Thus much, then, of the sanitary condition of the vicinity of stations and the localities in which they are most commonly situated. Of the defective condition of barracks and hospitals, the Report states as follows:—

"Both barracks and hospitals are built at or close to the level of the ground, without any thorough draught between the floors and the ground. And the men, both in barrack-rooms and sick-wards, are exposed to damp and malaria from this cause, as well as from want of drainage. The ventilation is generally imperfect; and from the arrangement of doors and windows, men are exposed to hurtful draughts. Many of the rooms are too high, and, as a consequence, there is much surface overcrowding both in barracks and hospitals, although with large cubic space. In a number of instances, both the space and area per bed are much too small. Barracks and hospitals have frequently no glazed windows, and only wooden shutters. Both barrack-rooms and sick-wards are, as a rule, dark. There are often four, or even six, rows of beds between the opposite doors or windows—increasing greatly the already existing difficulty of ventilation, and exposing the inmates to foul air. The greater proportion of the force is lodged in barracks, in such large numbers per room as to be very injurious to health; many of the rooms being several hundreds of feet in length, and some of them containing from a quarter to half of a regiment each!" (pp. 161-2.)

For the rest, the water-supply is most faulty—this being "a cardinal defect at Indian stations;" the means of personal cleanliness are insufficient, and often there are no baths; while in hospitals there is "no proper ablution or bath accommodation." The "means of cooking are primitive and imperfect—hardly suitable for permanent barracks, although the cooking is considered to be sufficiently varied;"—the "privies and urinals are generally of a bad or defective construction," unprovided with drains, and cleansed by manual labour; and the hospitals have "no water-closets, only open privies situated at a distance;" —the ration is complete, and of good quality, but "it is not varied to provide against the effects of the soldier's sedentary habits, and no difference is made for the cold and hot seasons;"

—the clothing is susceptible of being better adapted to the climate;—and, finally, the soldier is condemned during the greater part of the day to complete idleness—having no occupation except his military duties, and there is very insufficient provision for his recreation or instruction. As a consequence, intemperance and vice, with its loathsome consequences, are rife.*

It will be observed that the parallel between the sanitary condition of the Indian and the home army, prior to the Commission of 1857, is very close; and it is obvious that even in the temperate zone no body of men could live under such conditions as prevail in Indian stations except at the cost of great loss of life and physical vigour. This was fully demonstrated by the state of the army at home prior to the improvements effected by the investigation of 1857. Imagine, then, what the influence of insanitary conditions so marked must be upon health and life under the tropical sun. Heat and moisture are the two great agencies which have to be considered in estimating the effects of an Indian climate upon the system; and they are the two physical agencies most concerned in intensifying the morbid effects of defective sanitary arrangements. They are the main agents, moreover, in the production of the deadly malaria which is constantly present on the plains of India. Under the combined influence of the climatic, topographical, and insanitary conditions under which the soldier is placed in India, the diminution of efficiency in the army by sickness, invaliding, and death, is enormous. The annual death-rate throughout India since the commencement of the century has been no less than 69 per 1000 strength. From 1800 to 1830, the average was 84 per 1000; from 1830 to 1856, 57; and from 1850 to 1856, 50. The annual rate of invaliding is estimated at 44 per 1000; and the constant sickness rate at 84 per 1000. "With this amount of sickness, an army of 70,000 British in India has, so to speak, a vast hospital of 5880 beds constantly full of sick, and (taking the average mortality for the century) loses yearly by death 4830 men, or nearly five regiments."

At the present rate of mortality, both Sir Ranald Martin and Sir A. Talbot doubt whether it would be possible to maintain the proposed European establishment of 73,000 men. No less than 6037 recruits would be required to fill up the vacancies caused by death alone at an annual death-rate of 69 per 1000.

The parts played by the different morbific agencies which

---

* The Stational returns prepared for the Commission were submitted to Miss Nightingale. Her most valuable observations on the evidence contained in them, laid before the Commission, have been published in a separate handy form. Our readers would do well to procure this telling criticism on Indian hygiene.

have been enumerated, in causing this enormous loss of efficiency, and the probable result of well-conceived measures for diminishing it, are very clearly indicated by a further examination of the mortuary returns. It is highly significant of the most active sources of diseases, that the maladies chiefly destructive of the health and life of the British soldier in India are fevers, dysentery, and diarrhœa, diseases of the liver, and epidemic cholera. The influence of locality is shown by the fact that the death-rate varies from 20 per 1000 of the strength in the most healthy stations, to 115 per 1000 in the most unhealthy. The effects of defective sanitary arrangements are made known by the varied death-rates of men living under the same topographical, climatic, and, to a great extent, local conditions, but differently circumstanced as to certain particulars of personal and relative hygiene. Although the annual death-rate of the non-commissioned officers and privates of the Indian army is estimated at 69 per 1000, the annual mortality of the officers reaches but 38 per 1000, while that of civilians, of the soldier's age, distributed over the whole country, is but 20 per 1000. This last rate of mortality approximates to the highest average among the native troops, and the Commission adopts it as the standard of climatic and topographical influence upon the health of the European under comparatively favourable sanitary circumstances.

If, now, a comparison be instituted between the hygienic conditions to which the officer is subjected and that of the men, the most important difference observed is that of aggregation. Other not unimportant differences of personal hygiene could be named; but for our present purpose it is sufficient to point out that the officer is housed under conditions in themselves markedly less unhealthy, and less calculated to intensify the surrounding insanitary influences to which, in common with the privates and non-commissioned officers, he is exposed. The difference of mortality may be fairly taken as a criterion of the different degree in which the two classes of men are subjected to the local morbific influences which surround both in common. Again, the different rate of mortality between officers and civilians is to be elucidated precisely on the same grounds; and a like explanation may be largely extended to the differences existing between the death-rates of the European and native soldier. The sepoy has "an *instinctive horror of barracks,*" the Report states, "and retires from duty to the lines, where he finds his hut, into which not even the doctor dares to penetrate." (p. 44). Of the native lines it is said that they "constitute a very bad camp, in which every sanitary precaution is ignored, and the water is often very impure." In many stations the death-rate of the sepoys does not exceed 10 in

1000; and the excess observed above 10 is referred to malaria and the abominable sanitary conditions, apart from aggregation, under which he lives.

It would follow then, from these considerations, that the chief cause of the enormous mortality among the British forces in India is not the morbific influence of the climate as commonly understood, but the defective sanitary conditions under which the soldier is placed. That the climate does exercise a marked deteriorating effect upon the European constitution is fully admitted, but its influence is less direct than indirect.

"In the present state of Indian drainage and agriculture," says the Commission, "it is clear that for all practical purposes we must assume the three peculiarities we have enumerated—heat, moisture, and malaria—as constantly present and everywhere influencing the sanitary conditions of the country. These are ever tending to lower the general standard of health, and to predispose to epidemic diseases, but they are by no means the only causes in operation, although they exercise a potent influence on the comparative intensity of other causes. The presence of any or all of them, has the effect of rendering other causes of vastly greater importance than they would be *per se*. Negligence, or the absence of precautions, which in cooler climates would lead to little or no results, good or bad, become of great importance in India. A trifling degree of impurity of the air, brought about by a stagnant state of the atmosphere, or by overcrowding and want of ventilation in a barrack or station, may lead to a fatal outbreak of disease. Impurity in the water supply, such as would be attended with comparatively little influence on health in England, may, in India, determine an endemic attack of cholera or dysentery. Some apparently trivial inattention to cleanliness or drainage, of every-day occurrence at home, may lead to disastrous results where a number of men are crowded together on a small area." (p. 64.)

Assuming that the enormous mortality and sickness of the Indian army is principally due to the defective sanitary conditions under which the soldier is placed, it follows that these sources of inefficiency may be greatly diminished. The Commission is of opinion that—"The experience of the civil service, of the military officers, of their wives and children, of the English troops in many stations, and of the native troops, proves that in the present state of India the mortality of the English troops there can be reduced to the rate of 20 in 1000." To effect this great result the Commission (1) offers many suggestions, which it is not necessary to dwell upon, to meet the particular hygienic evils disclosed by the inquiry; and recommends (2) a fundamental change in the localization of stations and distribution of the troops, as well as (3) the organization of a complete system of sanitary administration.

Sir Ranald Martin, whose authority on all questions of tropical hygiene is supreme, submitted to the Commission a series of suggestions, which had also been laid before the Indian Government, on the advantages to be derived from cantoning British troops among the mountain ranges throughout India. It is undoubted that the climatic conditions of the elevated plains and hill ranges of India are much more favourable to the maintenance of the health of Europeans than those of the plains. Hitherto there has been no experience on a large scale of the sanitary influence of hill climates on healthy troops, and the whole subject requires to be submitted to a systematic investigation. But the general evidence derived from the physical condition of the hill-populations, and the particular evidence afforded by the hill-stations already existing and chiefly used as. sanitaria, and by the Lawrence asylums among the mountains (where European children grow up possessed of health as flush as is observed in England) are conclusive, apart from theoretical considerations, of the great superiority of the climate of the hills over that of the plains in the maintenance of health. Sir Ranald Martin holds that:—

"Nothing short of a proved necessity can warrant the condemnation of the British soldier to a residence on the hot and pestilential low grounds of India. It is," he says, "by reducing our garrisons on the plains to their minimum, by placing them in fieldworks open to the winds, in stations of proved salubrity comparatively, and by relieving them at the end of every year, and removing them for mental and bodily refreshment and invigoration back to the higher grounds, that their health and contentment may, in his view of the case, be preserved. Hitherto, under the ramparts of our old forts, and in our badly-selected and ill-arranged stations and open cantonments, our men have sunk away at the well-known rate already mentioned. . . . If the natural wants of the case, involving only a pure and cool air, with the means of exercise and amusement, be not anticipated, the moral and physical necessities of the European garrison will settle the question by the arrest of recruiting for India, and by the premature return of the limited service men, two rapidly exhausting causes, for the prevention of which the remedy is now at hand, if we would apply it. By persisting to confine our British soldiers to the dreadful monotony and endless miseries of barrack-life in the plains of India, we shall burn the candle at both ends, and leave the ultimate settlement of an important question, now very easy of being settled, to be concluded in a manner injurious to all authority." (*Evidence*, vol. i. p. 9.)

Every consideration, hygienic, economic, military, or political, tends to confirm these views. Hill stations would admit of the thorough training of recruits, without the deterioration of health experienced in the plains. They would also admit of a more efficient police, and consequent greater control over intemperance and venereal infection. Of the two last-named advantages, Sir Ranald Martin says, "Were there no considerations which recommend the selection of hill-stations other than those here referred to, I venture to say they would yet be paramount. If the mountain climates of India did not possess that which they are known to enjoy—namely, a pure, cool, and bracing air—still, I repeat it, the two considerations would and ought to give them a preference. The strength of the British army of India must be counted, not by the muster-rolls, but by its soundness of health, and its consequent real efficiency, both on moral and sanitary grounds." In the greater vigour and increased efficiency of the troops which would thus be derived from hill-stations, Sir Ranald Martin sees a gain which would more than counterbalance the presumed political evil of withdrawing the bulk of the garrison from the plains, and a surer security against outbreaks of insurrection. The ineradicable defects of many of the present stations on the plains, which must lead to their entire reconstruction, or to a change of locality, and the formation of new stations; the impossibility of placing native towns and existing bazaars in any satisfactory sanitary condition without a preliminary razing to the ground and destruction of the sub-soil; the absolute saving of life and vigour, looked upon as a question of pounds, shillings, and pence only, render it highly probable that the adoption of hill-stations would prove, even from the outset, the most economical as well as the best hygienic measure. The cardinal means of sanitary reform of the British army in India are, according to Sir Ranald Martin as follows :—

"(1) The careful selection of such stations on the plains as must, for political and military reasons, be occupied by Europeans, implying proper structural arrangements of every kind; (2) the occupation of table-lands by the artillery and cavalry; (3) the permanent occupation by the infantry of stations on the hill-ranges throughout our eastern possessions, for the preservation and maintenance of health; and (4) the selection of the best convalescent stations beyond sea, for the care of such invalids as may not regain their health in India. On this latter subject I would observe that, if the reports of the medical officers who have served in Western Australia be confirmed by future experiences, we shall there have attained a climate as nearly perfect as can be hoped for in any quarter of the globe.

"I would again beg leave to urge the establishment in our Indian armies of a well-supported sanitary department, separate and distinct from that ordered for the cure of disease and wounds. Personal hygiene, which must be left to the care of regimental surgeons and the medical staff of general hospitals, ought necessarily to be separated from the general military hygiene—the preserver of armies. Not to go further back, it was owing to the absence of a reliable and influential sanitary staff that our armies perished at Walcheren, at Rangoon, in China, and in the Crimea. It has been through the want of such officers as I have proposed to introduce into our armies that, in all our expeditions, and in all our foreign possessions, our soldiers have melted away, destroyed by sanitary neglect in our camps, by unwholesome stations, and by defective barrack and hospital accommodation; stations selected by ignorance, and which have been condemned at the first glance of experience by a qualified medical officer of health." (*Evidence*, i. p. 10.)

These weighty and prescient suggestions were adopted by the Commission, but it hesitated to give them, in respect to hill-stations, that complete development of which they were susceptible. The military evidence laid before the Commission seems to have influenced its conclusions. It was doubted whether the plains could be denuded of forces to so great an extent as Sir Ranald Martin recommended, with a due regard to the security of the country. The Commission advises that the strategic points on the alluvial plains should be reduced to a minimum, and that as few unhealthy stations should be held in force as possible. It further advises that a *third* of the force required to hold these points should be located on the nearest convenient hills or elevated plains, including in this third, by preference, men whose constitutions are becoming enfeebled, and recruits on their first arrival; and to give the other two-thirds their turn. If a system of annual relief were adopted, this would involve two years' residence on the plains and one on the mountains. Sir Ranald Martin's proposition is, that the bulk of the forces should be stationed on the hills, and that the garrison in the plains should be relieved annually. The sanitary aspect of the question admits of no doubt, while the military aspect is the subject of contending and irreconcileable opinions. It has not yet been determined what are the strategical points which require to be occupied in the plains, and until this "all-important element in the question," as the Commission designate it, is settled, it would perhaps have been well to avoid naming any limitation of the force to be stationed on the hills. Finally, the Commission advise that in the selection of hill-stations, simple elevation should never be trusted as a means of protecting health; but while the best available elevated stations should be occupied, these should be placed (as they require it just as much as the stations in the plains) in the very best sanitary condition.

The recommendations of the Commission respecting the formation of a complete system of sanitary administration, are best cited in detail:—

"There are, no doubt, considerable difficulties in the way of organizing an efficient sanitary service for India, and in adapting it to the various exigencies of the country; but there are, nevertheless, certain leading principles which should be kept in view in any administrative arrangements to be introduced for the purpose. It is, for example, of great importance that the procedure should be as far as possible uniform in each presidency; and this could be best secured by appointing commissions of health, one at each seat of Government, representing the various elements—civil, military, engineering, sanitary, and medical, on the co-operation of which depends the solution of many health questions. We are of opinion that such commissions are necessary also to give a practical direction to sanitary improvements and works.

"Their functions would be chiefly consultative and advising on all questions relating to the selection and laying out of stations, proper construction of barracks, hospitals, and other buildings, drainage, water-supply, cleansing, and general sanitary supervision in stations, cities, and towns, and on the prevention of epidemic diseases.

"To fulfil the other object of taking advantage of home experience, it would be necessary to afford these commissions every needful information on the most approved and economical methods of laying out sanitary works, and in those healthy principles of construction and improvement of barracks and hospitals which have been successfully carried out in England, but which have still to be introduced into India, and adapted to the circumstances of the country. The sanitary improvements which have been recently introduced at home military stations, and which are about to be carried into effect at certain foreign stations by the War Office, as well as the improved principles of construction in barracks and hospitals now in use, were adopted on the advice of a commission specially appointed by the War Office to inquire into the subject. The questions which arise out of the evidence from the Indian stations are of the same nature as those which have come under the examination of, and have been dealt with by, the War Office Commission; and it would be highly advisable to make their experience available for India by adding to the existing Commission an engineer and a medical officer conversant with Indian sanitary questions, or to form a similar commission in England for this object. Such a commission, if considered preferable, should include members specially conversant with recent improvements, military and civil, an engineer of Indian experience who has given attention to sanitary works, and a medical member acquainted with the sanitary question as it presents itself in India. The function of such a commission could of course be consultative only. It would simply be the medium of advising and informing the Indian Government and the presidency commissions on the latest improvements, and on the best principles of sanitary construction. For this purpose, it might give its advice on the healthiness or otherwise of plans, and as to the sanitary details of buildings to be occupied by troops; on the best and most economical methods of water-supply and drainage: it might collect and publish useful information and instructive matter regarding improvements, and it might possibly be able to give a more practical direction to the education of cadets of engineers destined for service in India, to enable them to devise works and improvements on healthy principles. It would in no way interfere with perfect freedom of action. It would place at the disposal of the Indian Government and presidency commissions the latest experience, classify and generalize the results of their several publications in a summary form, and thus enable all to arrive at a more satisfactory decision as regards measures to be carried out for protecting the health of troops than would otherwise be possible.

"As to the executive authorities by whom sanitary measures would have to be carried into effect, we apprehend that, as regards military buildings and stations, the powers should remain, as at present, with the Department of Public Works, whose plans and proposals would have to be submitted to the Presidency Commission for opinions on points affecting health.

"All plans of sanitary works and improvements, which might be proposed for native towns connected with stations, should also be submitted for advice and opinions to the Presidency Commissions.

"It would be advisable to begin this great work with the seats of Government, and to select a few of the more important stations to be thoroughly improved, as examples. This course would at once afford the necessary administrative experience, and a basis would be laid for future progress.

"Much time must necessarily elapse before much progress is made, but this we fear is inevitable." (pp. 158-60.)

The recommendations of the Commission further provide for the publication of a code of sanitary regulations specially adapted for India; the adoption in Indian stations of the system of army medical statistics in use at home stations; and the registration of deaths and the causes of death in the large cities of India.

There is but one doubt resting upon these recommendations, and that is, the form of sanitary government. Boards have not hitherto worked so well in India as to give any great confidence in the practical operations of a sanitary board. A board is a somewhat cumbersome agency, and it is scarcely in unison with the economy and discipline of the Indian army. An organization involving a greater amount of individual responsibility would probably have proved more effective in actual operation.

Apart from the objections noted—objections which are to be looked upon simply as indications of regret that the Commission did not feel itself justified in giving the recommendations upon which we have dwelt so full a development as they are capable of receiving, and which will, doubtless, follow in good time—the value of the conclusions and suggestions of the Commission cannot well be over-estimated. If the latter are carried out in their integrity, little doubt can be entertained that the results will be no less happy to the Indian army than those which followed in the army at home from the suggestions of the Royal Commission of 1857.

---

